# A Film Electrode upon Nanoarchitectonics of Bacterial Cellulose and Conductive Fabric for Forehead Electroencephalogram Measurement

**DOI:** 10.3390/s23187887

**Published:** 2023-09-14

**Authors:** Kunpeng Gao, Nailong Wu, Bowen Ji, Jingquan Liu

**Affiliations:** 1 The School of Information Science and Technology, Donghua University, Shanghai 200051, China; nathan_wu@dhu.edu.cn; 2 The Unmanned System Research Institute, Northwestern Polytechnical University, Xi’an 710072, China; bwji@nwpu.edu.cn; 3 Department of Micro/Nano-Electronics, Shanghai Jiao Tong University, Shanghai 200240, China

**Keywords:** EEG electrodes, wearable EEG, bacterial cellulose, conductive fabric, gel electrodes

## Abstract

In this paper, we present a soft and moisturizing film electrode based on bacterial cellulose and Ag/AgCl conductive cloth as a potential replacement for gel electrode patches in electroencephalogram (EEG) recording. The electrode materials are entirely flexible, and the bacterial cellulose membrane facilitates convenient adherence to the skin. EEG signals are transmitted from the skin to the bacterial cellulose first and then transferred to the Ag/AgCl conductive cloth connected to the amplifier. The water in the bacterial cellulose moisturizes the skin continuously, reducing the contact impedance to less than 10 kΩ, which is lower than commercial gel electrode patches. The contact impedance and equivalent circuits indicate that the bacterial cellulose electrode effectively reduces skin impedance. Moreover, the bacterial cellulose electrode exhibits lower noise than the gel electrode patch. The bacterial cellulose electrode has demonstrated success in collecting α rhythms. When recording EEG signals, the bacterial cellulose electrode and gel electrode have an average coherence of 0.86, indicating that they have similar performance across different EEG bands. Compared with current mainstream conductive rubber dry electrodes, gel electrodes, and conductive cloth electrodes, the bacterial cellulose electrode has obvious advantages in terms of contact impedance. The bacterial cellulose electrode does not cause skin discomfort after long-term recording, making it more suitable for applications with strict requirements for skin affinity than gel electrode patches.

## 1. Introduction

The electroencephalogram (EEG) is a technique used to monitor and record electrical activity in the brain. It can be used for a variety of applications, including diagnosing epilepsy and other neurological diseases; monitoring anesthesia; studying cognition and behavior; diagnosing sleep disorders; and monitoring brain activity [1,2,3,4]. The wearable devices have attracted much attention from both research institutions and technology companies [5,6,7,8], and information about health could be analyzed from the bio-potential recorded by them [9]. However, although EEG signals have been applied in medical diagnosis for decades, there are still some problems with the long-term monitoring of EEG [10]. Nowadays, EEG signals are usually recorded by wet electrodes in hospitals [11]. The wet electrode consisted of an Ag/AgCl or Au pad and conductive gel between the skin and the electrode pad. Although the wet electrode has low skin-electrode contact impedance and good signal quality, the complexity of its application and the difficulty of cleaning the conductive gel make it not suitable for multiple recordings. The electrode placement process is time-consuming and uncomfortable. In order to achieve convenient and fast EEG signal recording, the gel electrode has been widely applied in wearable devices [11]. The gel electrode is sticky and could adhere to the skin for a long time. Because of the conductive gel, the contact impedance is very low. However, there are still many drawbacks to these electrodes. The adhesiveness of gel electrodes could be affected by skin sweating. With the gradual increase in sweat secreted from the skin, the stickiness of the electrodes would decrease gradually. Moreover, the gel electrode patch needs to be fixed on the skin with viscose, which is not absorbent and breathable, and the viscose itself is also irritating to the skin. This causes the skin to become red, swollen, and itchy after continuous use of the gel electrode patch over a period of time. The adhesive used in the gel electrode is very sticky and easily sticks to hair, which can easily cause pain when the electrode is ripped off from the skin. These reasons make gel electrodes unsuitable for long-term, repeated use. Nowadays, many wearable dry electrodes have been researched [12,13,14,15]. The main research content is to record EEG in a hairy area [16,17,18]. However, the reference electrodes and other electrodes attached to the skin still mainly use uncomfortable gel electrode patches.

The main novelty of this paper is that the combination structure of the fabric electrode and bacterial cellulose film has better comfort while having a low impedance similar to that of the gel electrode. In previous designs, fabric electrodes were often used as dry electrodes. Although dry electrodes are convenient to use, the contact impedance of these electrodes is difficult to stabilize to low values due to the extremely high impedance of the skin surface. In this case, disposable gel patch electrodes are still widely used. However, the gel patches are not skin-friendly enough. The new electrode design in this article takes advantage of the skin affinity and water retention of bacterial cellulose. It can be adsorbed on the skin via a bacterial cellulose membrane and uses internal moisture to moisten the skin surface, providing low and stable contact resistance. Meanwhile, the bacterial cellulose membrane has a much higher skin affinity than the gel patch. In this way, the new electrode can be used to replace the widely used but easily uncomfortable gel patch electrodes.

Bacterial cellulose is a general term for cellulose. It is a kind of natural cellulose without any impurities [19,20,21]. Its properties include a fine network structure, high mechanical strength, water absorption, and water retention. Besides, bacterial cellulose also has many unique properties, such as good biocompatibility and biodegradability [22]. At present, bacterial cellulose is often used to make facial masks, with excellent skin affinity and adsorption. In this study, we propose a novel soft and moisturizing film electrode based on bacterial cellulose for EEG signal measurements. The bacterial cellulose electrode is composed of three layers: the bacterial cellulose membrane, the Ag/AgCl-coated conductive fabric, and the Eco-flex silicon rubber protective layer. The bacterial cellulose could absorb and retain moisture for a long time to reduce the skin-electrode contact impedance. Moreover, the bacterial cellulose is totally soft and can adhere to the skin through its own adsorption. The Ag/AgCl-coated conductive fabric [23] was pasted on the bacterial cellulose membrane, which could convert the ionic current on the skin to the electronic current in the lead wire. The Eco-flex silicon rubber is coated on the no-coverage side of the conductive fabric to prevent water evaporation. All three layers are fabricated from totally soft materials. The soft and self-adsorbing properties of the bacterial electrode could be pasted on active and uneven parts of the body. The testing results on the contact impedance of the skin electrode showed the bacterial cellulose had a similar contact impedance to disposable gel electrodes and even had a lower contact impedance per unit contact area. The quality of EEG signals recorded by the bacterial cellulose electrode is also tested. The result shows that its electrical properties and the collected EEG signals are very similar to those of the gel electrode patch. The experimental results show that the bacterial cellulose electrode could remain adhesive on the skin for a long time without damaging it.

The main contributions to this article include the following:

(1) A bacterial cellulose electrode structure is designed. This thin film electrode structure is completely flexible, retaining the water retention of the bacterial cellulose, which can be adsorbed on the skin, reducing the impedance between the skin and the conductive cloth electrode.
(2) A method for using bacterial cellulose membranes in a process requiring heating is proposed.
(3) The electrical properties of bacterial cellulose in collecting EEG signals are researched. The ability of bacterial cellulose electrodes to collect EEG signals is verified.

## 2. Materials and Methods

### 2.1. Design of the Electrode

**Figure 1:**
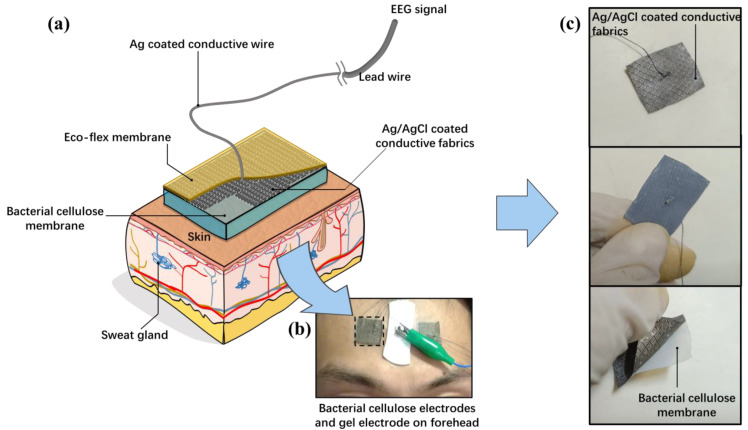
The structure of the bacterial cellulose electrode (**a**) The schematic diagram of the thin-film electrode structure (**b**) The bacterial cellulose electrodes (the two on either side) are pasted on the forehead beside a gel electrode patch (the middle one). (**c**) Layered structure of the electrode.

In this paper, bacterial cellulose is used to construct the base of the thin film electrode and retain water in it [24]. The purified cellulose network has many porous channels and can permeate water and air [25]. Figure 1 shows the structure of the bacterial cellulose electrode. The bacterial cellulose electrode is composed of three layers: the bacterial cellulose membrane, followed by the Ag/AgCl-coated conductive fabric, and the Eco-flex silicon rubber protective layer. The layer contacting the skin is the bacterial cellulose membrane. The bacterial cellulose is ultra-soft and could adhere to the skin due to the surface tension of water. The water in the bacterial cellulose could continuously permeate the skin and reduce the impedance of the cuticle. The back side of the bacterial cellulose film is covered by a waterproofing membrane to prevent water from evaporating into the air and provide the insulating layer for the electrode lead wire arrangement. When the bacterial cellulose-based electrode is attached to the skin, the soft film will adapt to the appearance of the skin surface. Sweat could be absorbed rapidly by the bacterial cellulose and supplement the moisture leaked from the edge of the film.

The conductive fabric coated with Ag/AgCl is pasted on the bacterial cellulose membrane. The conductive fabric is directly connected to the lead wire and converts the ionic current on the skin to the electronic current directly. Because the conductive fabric is also totally soft, it could be deformed synchronously with the bacterial cellulose membrane.

Eco-flex is a biocompatible and ultra-soft silicone rubber, and its Young modulus of elasticity is similar to that of skin. In our design, the Eco-flex film could prevent water evaporation into the air from the bacterial cellulose. The film could also prevent the conductive fabric from contacting the outside world.

The rehydration of bacterial cellulose directly affects the performance of electrodes. Under normal conditions, the completely dried bacterial cellulose could only absorb less than 1/3 of the moisture compared with the bacterial cellulose without drying. In this case, any processing method that requires vacuuming or high-temperature baking is unsuitable for bacterial cellulose treatment; the electrode fabrication process is applied at room temperature to avoid the complete dehydration of bacterial cellulose.

### 2.2. Electrode Fabrication

**Figure 2:**
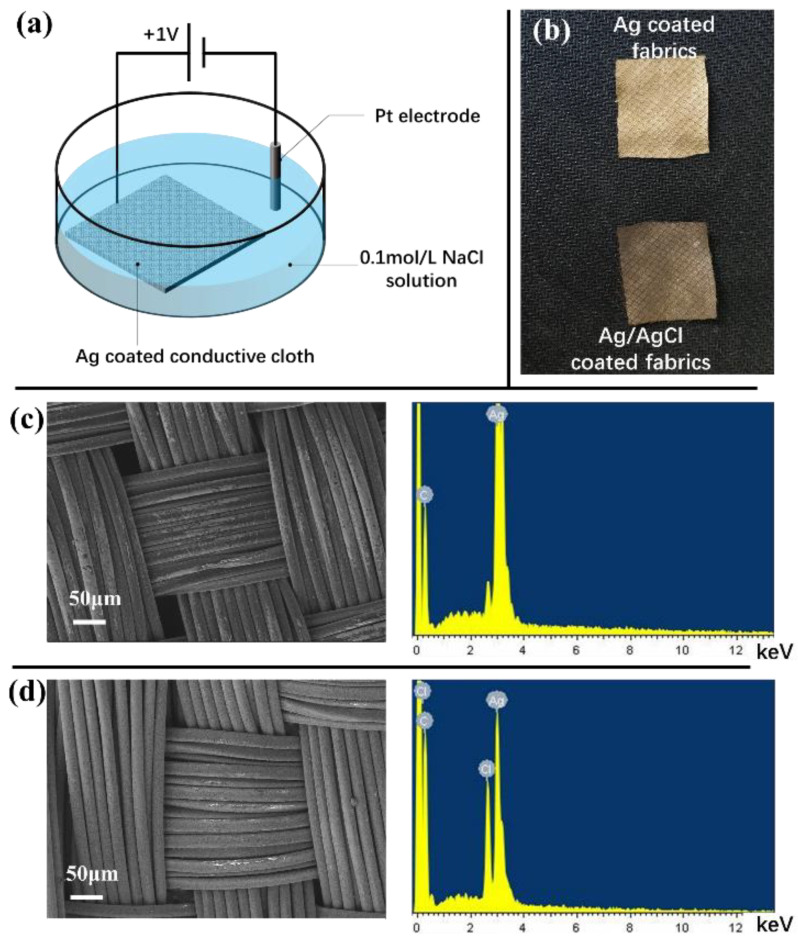
Ag/AgCl conductive cloth fabricated by Chlorination is the Ag-coated conductive fabric. (**a**) The schematic drawing of the chlorination method. (**b**) Conductive fabric before and after chlorination (**c**) Electron micrograph (right) and energy spectrum (left) of the conductive cloth without chlorination. (**d**) Electron micrograph (right) and energy spectrum (left) of the conductive cloth with chlorination.

Figure 2a shows the chlorination of Ag-coated fabric. The chlorination process is performed by a constant voltage method. The Ag/AgCl-coated conductive fabric is prepared from Ag-coated conductive fabric (SinoQ^®^ in Beijing, China). The Ag on the fabric is chlorinated by an electrochemical workstation (HC660). It is first immersed in a KCl solution with a concentration of 0.1 mol/L and connected to the work channel. Then, a Pt electrode is connected to the negative channel. A constant voltage with an amplitude of +1 V is applied between the working channel and the negative channel. The whole chlorination process lasts for 10 min.

The electrode is then cleaned with deionized water. Figure 2b shows the fabric before and after chlorination. Figure 2c,d show the coating on the fibers before and after chlorination under an electron microscope and the corresponding energy spectrum. A mass of Ag elements could be observed before chlorination, and a large number of Cl elements could be observed after chlorination. They indicate that an AgCl layer is deposited on the fiber surface.

**Figure 3:**
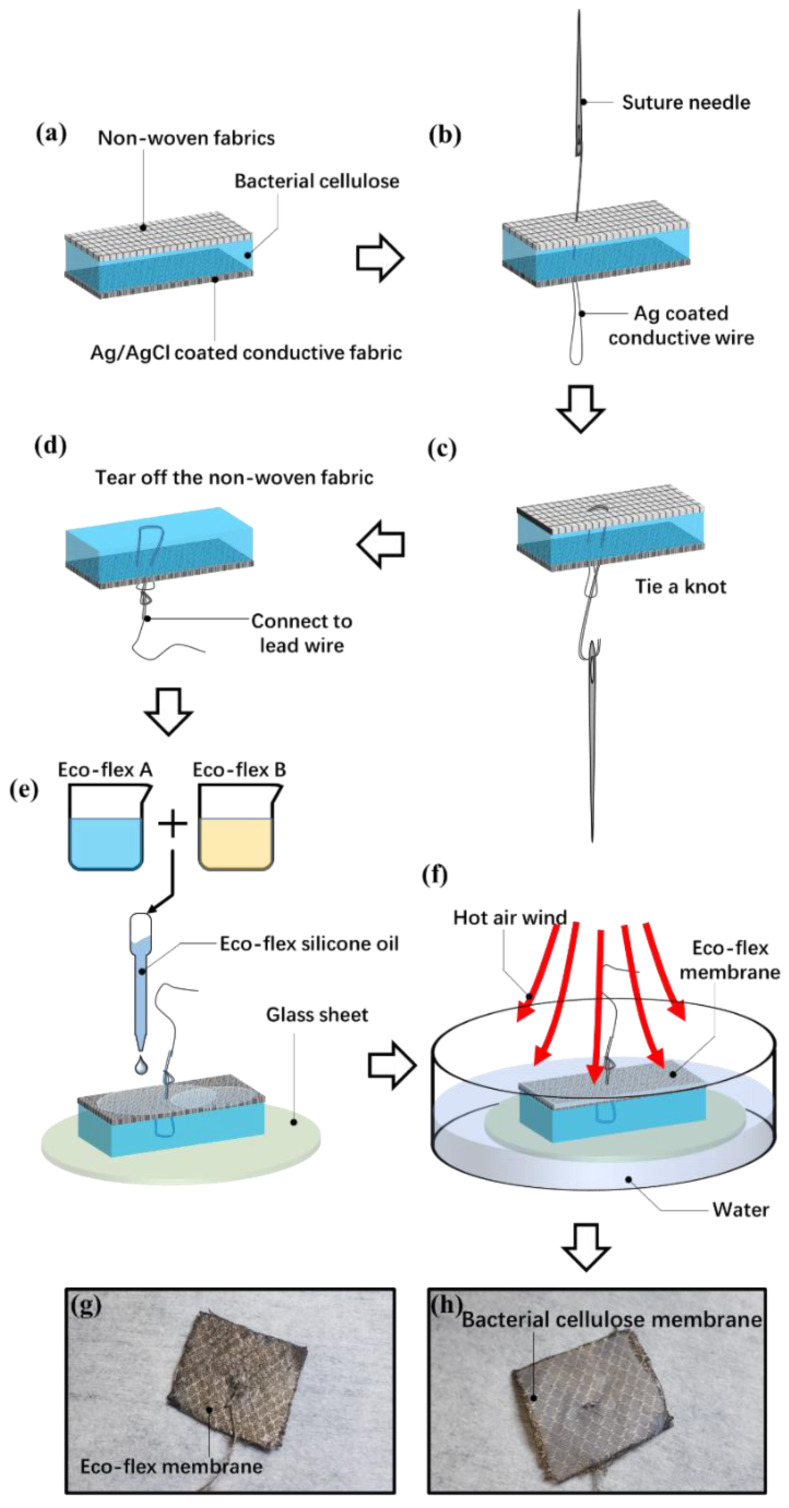
Fabrication process of the bacterial cellulose electrode and the assembled electrode. (**a**) Paste the bacterial cellulose film on Ag/AgCl-coated conductive fabric. (**b**) Seam the lead wire on the electrode. (**c**) Tie the knot. (**d**) Tear off the non-woven protective film. (**e**) Dispense Eco-flex silicone prepolymer. (**f**) Heating and curing the Eco-flex. (**g**) Conductive fabric side of the electrode. (**h**) Bacterial cellulose side of the electrode.

Figure 3 shows the fabrication process for the electrode. The bacterial cellulose membrane is pasted on the Ag/AgCl-coated fabric (Figure 3a). The two sides of the bacterial cellulose membrane are covered by non-woven fabrics. We tear off one side of the nonwovens first. A piece of dust-free paper is pasted on it to absorb the excess water in the bacterial cellulose. Then, the bacterial cellulose is pressed on the Ag/AgCl-coated conductive fabric. The air between the bacterial cellulose and the conductive fabric is driven out. Finally, the bacterial cellulose and conductive fabric are cut into 1.6 cm × 1.6 cm squares.

The lead wire is then connected to the bacterial cellulose membrane and Ag/AgCl-coated fabric. The wire is fabricated from Ag-plated nylon thread. The impedance of the wire is about 5–10 Ω/cm, and the weight is about 0.1 g/cm. The surface of the wire is then coated with Kafter K-704 electric insulating oil (Guangdong Evergrande New Material Technology Co., Ltd., Huizhou, China) to prevent short circuits and crosstalk. Because the conductive nylon wire has the characteristics of being light and extremely soft, it could prevent the pull on the electrode from the weight of the wire. Figure 3b–d show the connecting methods for lead wire. A needle is used to pass through the bacterial cellulose membrane and conductive fabric. Then, a knot is tied to prevent the lead wire from falling off.

After these procedures, the left piece of the non-woven fabric is torn off from the bacterial cellulose membrane. The bacterial cellulose is then pasted to a glass sheet. The conductive fabric side of the electrode is then covered with Eco-flex silicone rubber to prevent the evaporation of water (Figure 3e). The Eco-flex is composed of two oily components, which are first mixed at a mass ratio of 1:1. Then, the oily Eco-flex oil is drip-coated on the Ag/AgCl-coated conductive cloth. The whole electrode and glass sheet are put into water, as Figure 3f shows. The Eco-flex rubber was heated by hot air. The wind temperature is set at 130 °C. The water could prevent the bacterial cellulose from overheating. Figure 3g,h show the final state of the electrode. One side is covered with bacterial cellulose to stick to the skin. The other side is covered with an Eco-flex membrane.

## 3. Results

**Figure 4:**
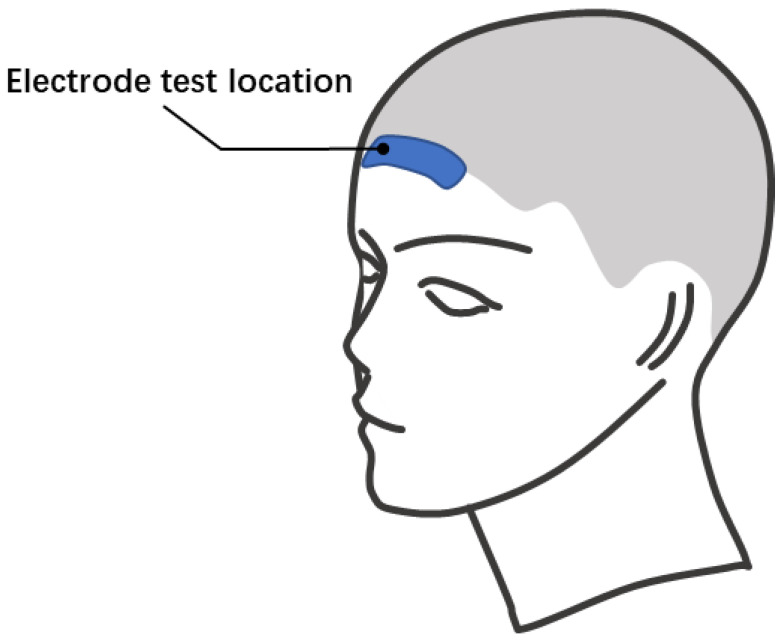
The electrodes’ positioning layout during impedance measurement and EEG signal measurement.

To evaluate the performance of the bacterial cellulose electrode, the bacterial cellulose electrode, and the 3M2223CN gel electrode are tested and compared. Both water and physiological saline are used as the electrolytes in bacterial cellulose electrodes. The different ion concentrations in water and brine simulate the effect of chloride ion loss on electrode performance with use. Figure 4 shows the electrode positioning layout during impedance measurement and EEG signal measurement. The experiment in this paper has been approved by the Scientific Research Ethics Committee of Bio-x Center, Shanghai Jiao Tong University. All the subjects knew the test procedure and were completely voluntary.

### 3.1. Contact Impedance on Different Subjects

The skin-electrode contact impedance on different subjects and locations is measured to evaluate the performance of recording bio-potential signals. In this section, the skin-electrode contact impedance is measured by the impedance measure function of the NuAmps EEG recording system (Neuroscan Pty. Ltd., SEA. Charlotte, NC, USA). Two bacterial cellulose electrodes and a 3M2223CN gel electrode are tested on the forehead. During the measurement, the GND electrode is a wet electrode, which is an Au cup electrode with Ten 20 conductive gel (Weaver Company, Lehi, UT, USA), whose contact impedance is lower than 5 kΩ. The GND electrode is pasted next to the tested electrode.

**Figure 5:**
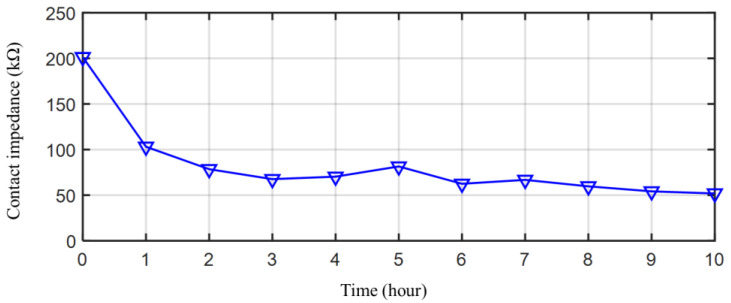
The contact impedance of Ag/AgCl conductive fabric dry electrode without bacterial cellulose on it.

**Figure 6:**
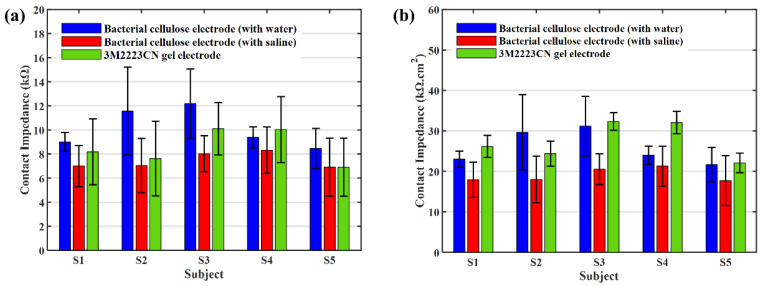
The contact impedance test results on different subjects of bacterial cellulose electrodes and gel electrodes (**a**) Contact impedance and the error bars of different electrodes. (**b**) Normalized contact impedance and the error bars of different electrodes.

Before the test of the bacterial cellulose electrode, its Ag/AgCl conductive cloth is tested. Simple conductive fabric electrodes have a high contact impedance. Figure 5 shows the contact impedance of an Ag/AgCl conductive fabric dry electrode without bacterial cellulose on it. With the secretion of sweat, the contact impedance of the conductive cloth drying electrode decreased gradually; however, the contact impedance was higher in the dry state. And its contact impedance is always much higher than that of a gel electrode. At this time, the dry electrode can collect EEG signals; however, the signal quality is lower than that of the patch gel electrode [26]. Figure 6a shows the contact impedance. They are the average of the five measurements. The error bars are the standard deviations of the measured values.

The contact impedance is then normalized by dividing by electrode area. The size of the bacterial cellulose electrode is about 1.6 cm × 1.6 cm, and the square is about 2.56 cm^2^. The 3M2223CN gel electrode is composed of a 9 mm-diameter Ag/AgCl round pad and a 2 cm × 1.6 cm rectangle conductive gel pad. The foam backing of the gel electrode is a round pad with a diameter of 4.3 cm. The effective contact square of a gel electrode is equal to the size of the conductive gel. The square of the conductive gel is about 3.2 cm^2^. Figure 6b shows the normalized skin-electrode contact impedance.

The contact impedance of the bacterial cellulose electrode is very similar to that of the 3M2223CN gel electrode, although the area occupied on the skin is much smaller (the foam backing of the gel electrode occupies about 14.5 cm^2^, while the bacterial cellulose electrode occupies only 2.56 cm^2^). According to Figure 6b, the contact impedance per unit area of a bacterial cellulose electrode is lower than that of a 3M2223CN electrode. It indicates that the bacterial cellulose electrode may have better conductivity than the gel electrode.

### 3.2. Contact Impedance under Different Frequencies

**Figure 7:**
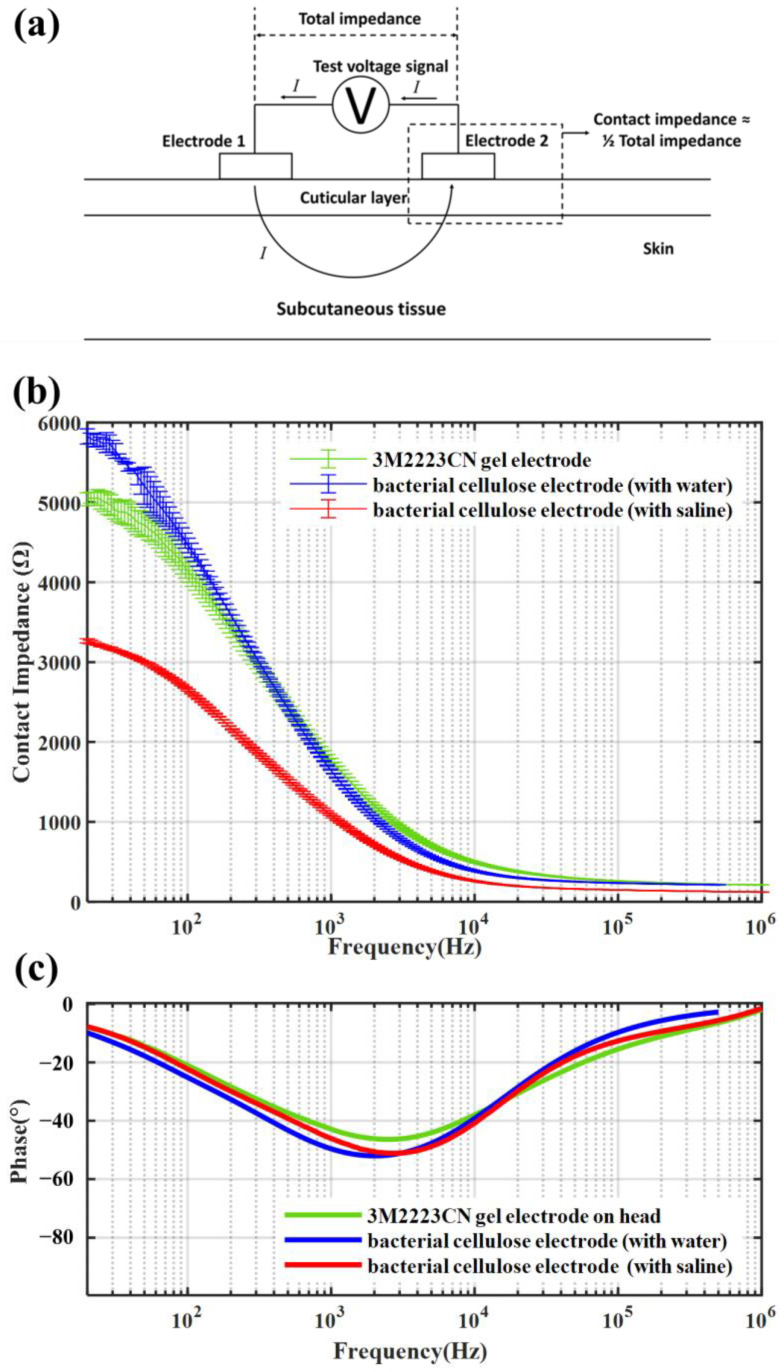
The test method and results of contact impedance and phase for the bacterial cellulose electrodes and gel electrodes are compared. (**a**) The schematic diagram of the two-electrode method for contact impedance measurements. (**b**) The comparison of contact impedance at different frequencies. (**c**) The comparison of phase at different frequencies.

The contact impedance for different signal frequencies is measured with a Keysight E4990A impedance analyzer (Keysight Technology (China) Co., Ltd., Shanghai, China). The amplitude of the test signal is 1 mV. The frequency of the excitation signal ranges from 20 Hz to 1 MHz. The contact impedance is measured by the two-electrode method. Figure 7a shows the testing method. During the test, two identical electrodes are pasted on the skin nearby, and an AC voltage signal is applied between them. The impedance between two electrodes could be measured by the current flowing through the electrodes. Because the impedance of subcutaneous tissue is significantly less than the electrode-skin interface impedance, it could be ignored in contact impedance measurement. Then, the skin-electrode contact impedance could be considered half of the total impedance between two electrodes. We test the contact impedances on the forehead. Figure 7b shows the contact impedance of different electrodes. They are the average of the five measurements. The error bars are the upper and lower limits of the measured values. The result shows the bacterial cellulose electrodes have similar skin-electrode contact impedances compared with gel electrodes. When the signal frequency increased, all the electrodes had a decrease in contact impedance, and they varied in a highly consistent manner. Compared with the gel electrode, the contact impedance of the bacterial cellulose electrode reduces faster, which indicates less attenuation of high-frequency signals. Figure 7c shows the phase of both electrodes. The phase changes of the three electrodes are highly consistent. Therefore, the bacterial cellulose electrode meets the performance requirements for replacing the gel electrode in the signal phase.

### 3.3. Equivalent Circuit

**Figure 8:**
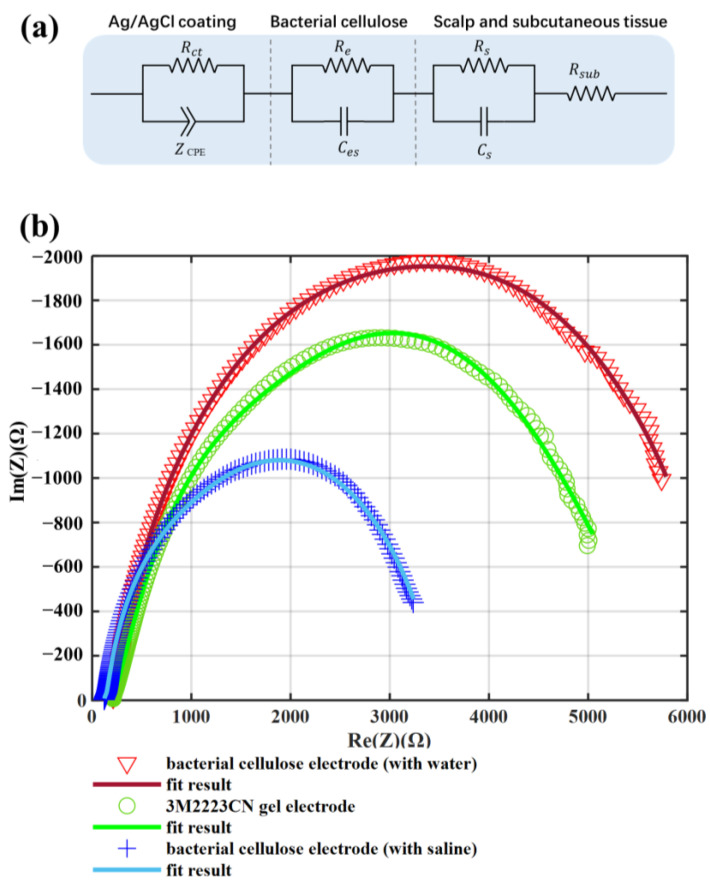
The equivalent circuit test result for EEG electrodes. (**a**) Equivalent circuit model of a gel electrode and a bacterial cellulose electrode on the skin. (**b**) Nyquist plot of gel and bacterial cellulose electrodes on the head.

The equivalent circuit is tested on the forehead, calculated from the Nyquist diagram by Zview2 (3.0.0.22, Scribner Associates, Inc., Southern Pines, NC, USA). Figure 8a shows the equivalent circuit model for both bacterial celluloses and gel electrodes [27,28,29]. The model included the impedance between the electrode pad and skin, the impedance of the electrode itself, and the impedance of skin and subcutaneous tissue. Because the bacterial cellulose is a kind of hydrogel, the electrode fabricated from it could have a similar impedance model structure as a gel electrode. *R_e_* and $C_{es}$ represents the resistance and capacitance between electrolyte and electrode, respectively. The $R_{ct}\;$represents the charge transfer resistance. The skin consisted of several layers that could be modeled by $R_{s}$ and $C_{s}$ in parallel. The $R_{usb}$ represents the resistance of the body except the skin. The $Z_{CEP}$ represents the interface of electrode and electrolyte. The constant phase element (CPE) is expressed as follows:

(1) $$Z_{CEP}=\frac{1}{Y_{0}{\left(j\omega \right)}^{n}}\;$$

where $j=\sqrt{-1}$, ω represents the angular frequency $\left(rad\;s^{-1}\right)=2\pi f$, and f is the frequency in Hz. The parameter n changes from 0 to 1, corresponding to the CPE changing from a pure resistance to a pure capacitance. The parameter $Y_{0}$ represents the capacitance value when n = 1.

Figure 8b shows the Nyquist diagram of a bacterial cellulose electrode and a 3M2223CN gel electrode. Table 1 shows the parameters of an equivalent circuit. The *n* value in the bacterial cellulose electrodes and gel electrodes is basically the same. It means that the deformation of the EEG signal through the electrode is basically the same. The impedance of *R_e_* and $C_{es}$ in bacterial cellulose electrodes is significantly lower than that in gel electrodes. It may be due to the large effective area of the Ag/AgCl conductive fabric in the bacterial cellulose electrode and the good skin adaptability of the bacterial cellulose film. These results show that the bacterial cellulose electrode can replace the gel electrode without changing the signal frequency composition while having a lower contact impedance.

### 3.4. Noise of the Electrodes

**Figure 9:**
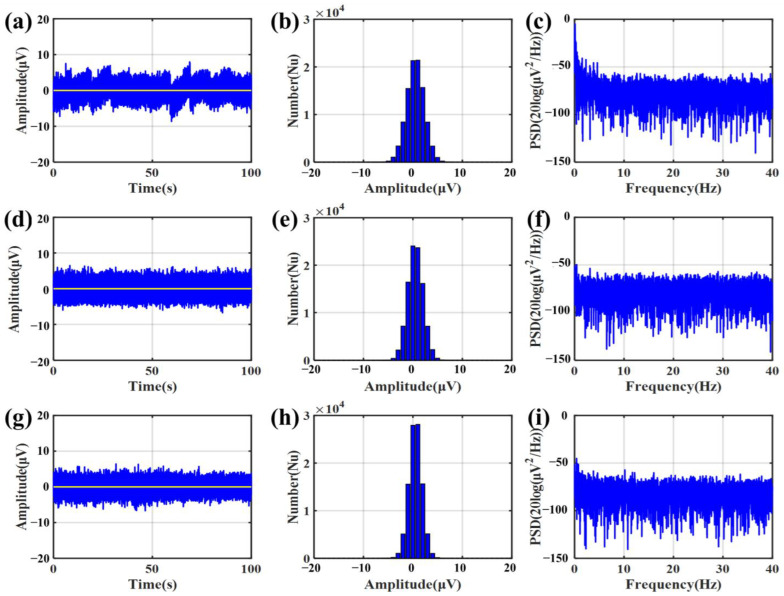
Short-circuit noise of the bacterial cellulose electrode and gel electrode. (**a**) Noise measured by gel electrodes. (**b**) Number of noises measured by gel electrodes with different amplitudes. (**c**) PSD of noises measured by gel electrodes. (**d**) Noise measured by bacterial cellulose electrodes (water as electrolyte). (**e**) Number of noises measured by bacterial cellulose electrodes with different amplitudes (water as electrolyte). (**f**) PSD of noises measured by bacterial cellulose electrodes (water as electrolyte). (**g**) Noise measured with a bacterial cellulose electrode (physiological saline as electrolyte). (**h**) Number of noises measured with a bacterial cellulose electrode with different amplitudes (physiological saline as electrolyte). (**i**) PSD of noises measured with a bacterial cellulose electrode (physiological saline as electrolyte).

When measuring the short-circuit noise, three bacterial cellulose electrodes are pasted on a polished silver sheet. The noise signals are measured by a NeuroScan NuAmps amplifier (NeuroScan^®^ NuAmpsNeuroscan Pty. Ltd., SEA. Charlotte, NC, USA). To avoid power frequency noise, one of the signal recording channels is selected as the reference channel. The noise signal could be considered the signal from the recording channel subtracted from the signal from the reference channel. After testing the short-circuit noise of the bacterial cellulose electrode, the electrodes were removed from the silver sheet. Three 3M2223CN gel electrodes are pasted on it and tested with the same method. Figure 9 shows the short-circuit noise of bacterial cellulose and gel electrodes. The result showed the bacterial cellulose electrode had a slightly lower noise level compared with the gel electrode. In the bacterial cellulose electrodes, saline has lower noise than water. Both saline and water have lower noise than gel electrodes. Therefore, the self-noise of the bacterial cellulose electrode is stable and reliable.

### 3.5. EEG Signal Measurement

The bio-potential signals are also measured by the NeuroScan NuAmps amplifier (NeuroScan^®^ NuAmpsNeuroscan Pty. Ltd., SEA. Charlotte, NC, USA). During the EEG signal measurement, two bacterial electrodes and one 3M2223CN gel electrode are pasted on the forehead. The extra foam quilt of 3M2223CN electrodes is cut off to reduce the distance between electrodes to about 1 cm. The reference and GND electrodes are wet electrodes (an Au cup electrode with Ten 20 conductive gel (Weaver Company, Lehi, UT, USA)). Because the alpha rhythm has very distinct frequency characteristics (a peak around 10 Hz on the power spectral density (PSD) of the EEG signal), we use it to evaluate the performance of bacterial cellulose electrodes in detecting EEG signals. The subject is asked to open or close his eyes alternately.

**Figure 10:**
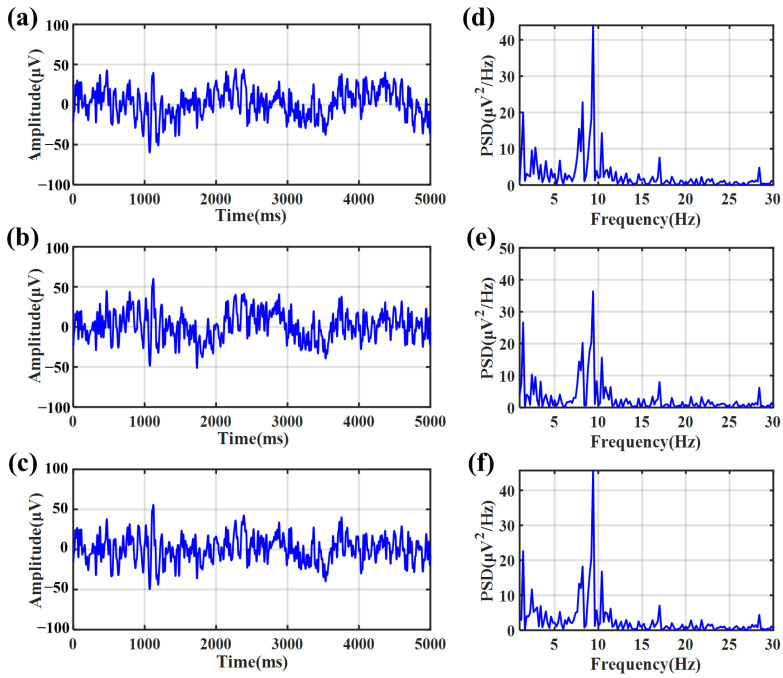
EEG signals recorded with eyes closed. (**a**) EEG signal recorded by a bacterial cellulose electrode with drinking water as an electrolyte. (**b**) EEG signal recorded by bacterial cellulose electrode with physiological saline as electrolyte. (**c**) EEG signal recorded by a bacterial cellulose electrode with gel electrolyte. (**d**) PSD of the EEG signal recorded by a bacterial cellulose electrode (drinking water as electrolyte). (**e**) PSD of the EEG signal recorded by a bacterial cellulose electrode (physiological saline as electrolyte). (**f**) PSD of the EEG signal recorded by the gel electrode.

**Figure 11:**
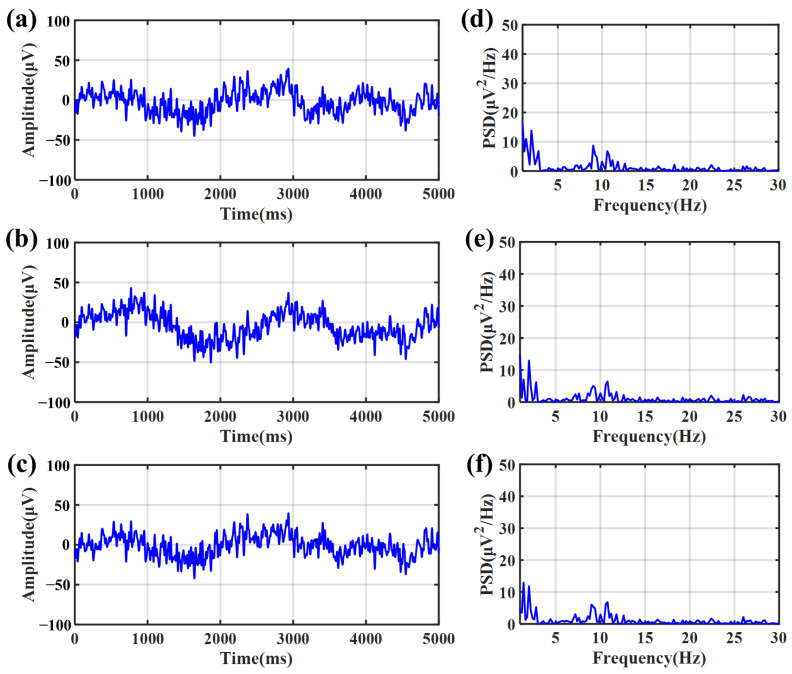
EEG signals recorded with eyes opened. (**a**) EEG signal recorded by a bacterial cellulose electrode with drinking water as an electrolyte. (**b**) EEG signal recorded by bacterial cellulose electrode with physiological saline as electrolyte. (**c**) EEG signal recorded by a bacterial cellulose electrode with gel electrolyte. (**d**) PSD of the EEG signal recorded by a bacterial cellulose electrode (drinking water as electrolyte). (**e**) PSD of the EEG signal recorded by a bacterial cellulose electrode (physiological saline as electrolyte). (**f**) PSD of the EEG signal recorded by the gel electrode.

Figure 10 shows the EEG signals recorded with the eyes closed. The signals recorded by the bacterial cellulose electrode with water (Figure 10a) as the electrolyte, saline (Figure 10b) as the electrolyte, and the gel electrode (Figure 10c) are quietly similar visually. Figure 10d–f shows the PSD of three signal channels. In the PSD, the peak at the frequency of alpha rhythms’ signal bandwidth from 8 to 13 Hz could be clearly observed. Figure 11a–c show the EEG signals recorded with the eyes opened. When the eyes were kept open, the trace of alpha rhythms disappeared in the PSD, as shown in Figure 11d–f.

In Figure 10 and Figure 11, the PSD recorded with eyes closed or opened shows the bacterial cellulose electrodes have a similar distribution compared with the 3M2223CN gel electrode in EEG signal measurement, whether the electrolyte in the bacterial cellulose is water or saline. This phenomenon suggests that the bacterial cellulose electrode and the 3M2223CN gel electrode had similar performance in the measurement of EEG signals. And it suggests that the electrode’s performance is insensitive to ion concentrations within bacterial cellulose and can provide sufficient stability for EEG signal recording.

### 3.6. The Signal Coherence of Different Electrodes

**Figure 12:**
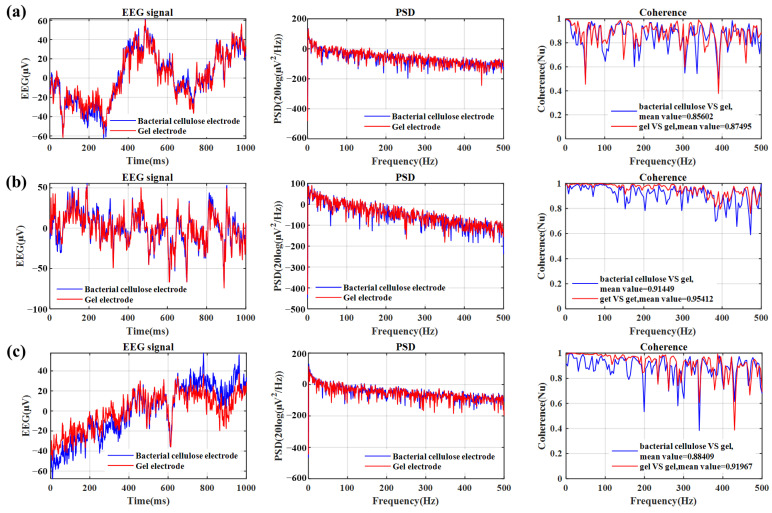
EEG signal recording (right), PSD (middle), and coherence (left) are compared between the bacterial cellulose electrode and the gel electrode. (**a**–**c**) correspond to different subjects.

To quantify the similarity of EEG signals recorded by a bacterial cellulose electrode and a gel electrode, we use both electrodes to collect EEG signals in the forehead area simultaneously. Three subjects (one female and two males) were tested for EEG signals. The two gel electrodes are placed on both sides of the bacterial cellulose electrode. The average of the two gel electrodes is compared with the bacterial cellulose electrode. As can be seen from the comparison results in Figure 12, the coherence of the two signals indicates that the EEG signals collected by different electrodes are very close. Especially in the range of the major EEG components of 0–100 Hz, the two are extremely close and maintain a high value. The average coherence between the bacterial cellulose electrode and the gel electrode is 0.86, while the average coherence between the two gel electrodes is 0.89, indicating that the device has a very similar EEG recording ability to the gel electrode. When the electrodes are removed from the skin after tests, the subject in Figure 12b reports that the gel electrode is stuck to the hair and is very painful. In response, all subjects reported that the bacterial cellulose electrodes had little or no sensation on the skin.

### 3.7. The Effect on the Skin

**Figure 13:**
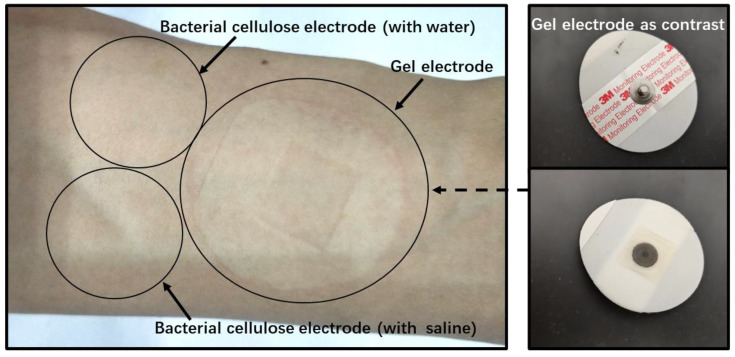
Damage to the skin from bacterial cellulose electrodes and gel electrodes: the gel electrode left obvious red and swollen marks on the skin and caused obvious itching of the skin.

To evaluate the effects of BCI devices on the skin, we attached two bacterial cellulose electrodes (using water and saline as electrolytes, respectively) and a 3 M2223CN gel electrode to the skin. The test is performed on the inner forearm. All electrodes are left on the skin for one hour and then removed. Figure 13 shows the skin condition after the electrodes are removed. Among them, the gel electrode left obvious red and swollen marks on the skin and caused obvious itching. After the removal of two bacterial cellulose electrodes, almost no visible traces are left, and there is no skin discomfort.

### 3.8. Life Span

Although the conductive Ag-coated electrode can be reused, the bacterial cellulose itself is not suitable for multiple uses. Over time, the soft bacterial cellulose will gradually deform. At the same time, the sweat also seeps into the bacterial cellulose. Given the extremely low cost of a single electrode (less than $0.01 per piece), we recommend using a new electrode each time.

**Figure 14:**
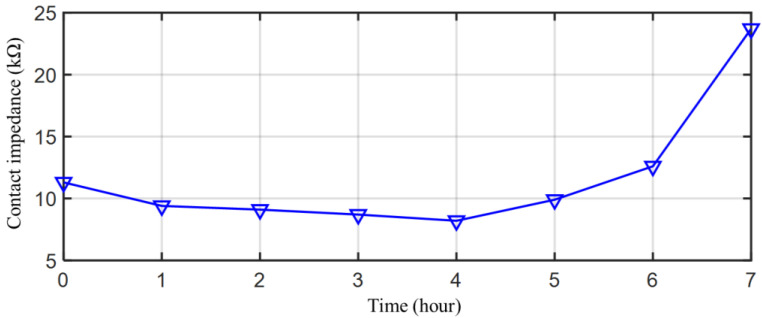
Life span of the bacterial cellulose electrode for a single EEG acquisition.

For a single EEG acquisition, the recording duration was mainly affected by the rate of water loss from bacterial cellulose in the electrode. With use, the water in the electrode is gradually lost. When the moisture loss rate reaches a limit, the electrode loses its low-impedance contact with the skin. To evaluate the duration, the contact impedance of a bacterial cellulose electrode is measured for 7 h. Figure 14 shows the life span of the bacterial cellulose electrode for a single EEG acquisition. After about 6 h, the contact impedance increases dramatically and you lose effective contact with the skin. The life span of the bacterial cellulose electrode for a single EEG acquisition can be considered to be 6 h.

## 4. Discussion

As the test of contact impedance shown in Figure 6 shows, the bacterial cellulose electrodes, which absorb saline or water, both have lower contact impedance than the gel electrode patch. It means that the bacterial cellulose electrode can achieve a stable low contact impedance, even if the electrolyte is gradually lost during use. The bacterial cellulose electrodes and the gel electrode patch are all coated with Ag/AgCl. The Ag/AgCl coating is the interface that transforms the signal of ionic current in gel into electron current in metal. Compared with the bacterial cellulose electrode, the Ag/AgCl pad in the gel electrode is much smaller. Although the gel pad has a larger area than the bacterial cellulose membrane. A large proportion of ion current signals need to be transmitted over a long distance in the gel pad to reach the Ag/AgCl pad. It increases some contact impedance. In terms of the fitting data in Table 1, it appears as a larger $R_{e}$. On the other hand, this larger transmission distance also reduces the equivalent capacitance between both ends of this section of material, as shown by a much smaller $C_{es}$ in Table 1.

Compared to bacterial cellulose membranes, the gel in the electrode patch contains less free water; therefore, it is less effective at wetting the top layer of the skin. Correspondingly, the skin under the gel electrode patch has a higher impedance. It behaves as a larger $R_{s}$ in the gel electrode patch than the bacterial cellulose electrodes. Because the gel is harder than the bacterial cellulose film, it will also fit less tightly to the skin than the bacterial cellulose film. It results in a smaller $C_{s}$ in the gel electrode patch, which corresponds to the equivalent capacitance of the outermost layer of skin.

As Figure 2d shows, the Ag/AgCl coating is evenly distributed on the fiber surface. It leads to a much larger equivalent area for ion exchange than the Ag/AgCl pad in the gel electrode patch. Therefore, it can bring a smaller equivalent resistance and a larger equivalent capacitance to the double layer. It shows that the $R_{ct}$ of the bacterial cellulose membrane is smaller than that of the gel electrode patch. And the $Y_{0}$ of the bacterial cellulose electrode with saline in it is larger than that of the gel electrode. As a comparison, the bacterial cellulose electrode with water in it has a much larger $Y_{0}$, which is because there is a lack of chloride ions in the water to complete the ion exchange. In connection with this, the bacterial cellulose electrodes show lower noise than the gel electrodes. The noise is mainly affected by the Ag/AgCl material on the electrode surface. The Ag/AgCl in the bacterial cellulose electrode is obtained by an anodic chlorinated Ag coating, which completely covers the Ag coating. The Ag/AgCl coating of the gel electrode is usually obtained by applying Ag/AgCl paste, which has lower uniformity. Correspondingly, the ion exchange on its surface tends to be more unstable, which leads to a larger noise than that of a bacterial cellulose electrode.

At present, the novel comfortable EEG electrodes mainly include dry conductive rubber electrodes [30], conductive fabric electrodes [26], gel electrodes [31], and temporary tattoo electrodes [32]. The rubber electrode is a dry electrode that uses the flexibility of rubber to adapt to the shape of the skin’s surface. The structural strength of the pure dry electrode is very durable and can be used multiple times without damage. However, the dry electrode is difficult to overcome due to the impedance of the cuticle of the skin, which makes its contact impedance often high, above 50 kΩ·cm^2^ [30]. Moreover, rubber dry electrodes often require additional fixing measures to attach to the skin, which brings some inconvenience to their use.

The conductive fabric electrodes are usually fabricated from Ag-conductive cloth. The fabric electrode is soft and breathable. Therefore, this type of electrode has good comfort. However, similar to the rubber electrode, the fabric electrode cannot reduce the impedance of the skin cuticle; therefore, the contact impedance is also much higher than the gel electrode patch, around 200 kΩ·cm^2^ [26].

Therefore, the gel electrode patch is still mainstream in practical applications. In this paper, we test a commercialized 3M2333 electrode patch, which exhibits low contact impedance and strong adhesion. However, the gel patch is not breathable, and the glue on it is easy to cause skin allergies or dermatitis. The temporary tattoo electrodes adhere to the skin by van der Waals forces. It is very soft, which can improve the comfort of the EEG collection. Its contact impedance is around 17 × 10^3^ kΩ·cm^2^ [32]. Moreover, it is a disposable electrode. Therefore, it is also not suitable to replace gel electrode patches.

In this paper, we combined the fabric electrode with bacterial cellulose film. It has the water absorption and breathability of both conductive fabric and bacterial cellulose. Moreover, the bacterial cellulose membrane that comes into direct contact with the skin is a biocompatible material. Therefore, it will not cause skin discomfort even if it is used for a long time. Because bacterial cellulose is flexible and water-retaining, it can be gently attached to the skin. Moreover, the bacterial cellulose can continuously moisten the skin surface, therefore the contact resistance between the electrode and the skin can be reduced to a slightly lower level than a gel electrode patch. Its contact impedance is about 19 kΩ·cm^2^. Table 2 shows the comparison.

The main purpose of designing this bacterial cellulose electrode is to improve comfort while maintaining the same signal quality as the disposable patch electrode. In this case, the bacterial cellulose electrode improves comfort while retaining some of the shortcomings of the gel electrode patch. Similar to gel electrode patches, this bacterial cellulose electrode is not suitable for repeated use. This is a challenge to the improvement of the bacterial cellulose electrode.

Another limitation of the bacterial cellulose electrode is that it is less sticky than the glue on the patch electrode. This means that in some applications where pulling forces are high, such as during exercise, the bacterial cellulose electrode may require additional holding measures, such as a head cover or bandage.

Although bacterial cellulose exhibits good biocompatibility and biodegradability, there is a potential risk of allergies or adverse reactions in certain individuals. When using the bacterial cellulose electrode, assessing and monitoring individual allergic reactions is needed. In addition, some bacterial cellulose allergies are caused by other impurities inside the bacterial cellulose [28,29]. Although bacterial cellulose itself has a small amount of antibacterial activity, there will still be bacterial growth. Therefore, the use of bacterial cellulose electrodes should be kept clean and hygienic, and the contaminated electrodes should be replaced in time.

## 5. Conclusions

In this paper, a soft and moisturizing film electrode based on bacterial cellulose is presented for bio-potential recording. The electrode is fabricated entirely from flexible materials. The electrode could be expediently adhered to the skin by the bacterial cellulose membrane. The water in bacterial cellulose could continuously moisten the skin and reduce the contact impedance. The skin-electrode contact impedance of a bacterial cellulose electrode is usually less than 10 kΩ, which is less than a commercial gel electrode, which is the target being replaced. The noise test showed the bacterial cellulose electrode has good signal quality. When recording EEG signals, the bacterial cellulose electrode and gel electrode have an average coherence of 0.86, which means that they all have similar performance across different EEG bands. And compared with gel electrode patches, the bacterial cellulose electrodes do not cause skin discomfort when worn for a long time.

Because of its excellent biocompatibility and comfort, the bacterial cellulose electrode has the potential to be applied to the EEG recording of infants and children or long-term EEG measurements. These applications have strict requirements for the skin affinity of the electrodes, and ordinary gel electrode patches are easy to cause dermatitis with. The ordinary dry electrode has a higher impedance and is not suitable for replacing the gel electrode. Moreover, the adsorption of the bacterial cellulose electrode allows it to be flexibly placed on the skin surface.

For now, the bacterial cellulose electrodes are still disposable. In order to improve the bacterial cellulose electrode in the future, the doping of bacterial cellulose can be further studied. Doping can improve the water retention and adsorption capacity of bacterial cellulose so that the electrode can withstand repeated use.

## Figures and Tables

**Table 1:** The numerical fitting results of equivalent circuit components of wet electrodes.

Item	$R_{e}$	$C_{es}$	$Y_{0}$	*n*	$R_{ct}$	$C_{s}$	$R_{s}$	$R_{usb}$
Unit	Ω	F	$\Omega^{-1}\cdot {cm}^{-2}\cdot s^{n}$	Nu	Ω	F	Ω	Ω
Gel electrode	89.24	1.65 × 10^−8^	1.11 × 10^−6^	0.757	4508	1.83 × 10^−7^	724.1	157.3
Bacterial cellulose electrode (water)	10.22	8.89 × 10^−8^	10.79 × 10^−6^	0.769	5480	2.69 × 10^−7^	693.4	208.9
Bacterial cellulose electrode (saline)	18.2	1.0 × 10^−7^	1.52 × 10^−6^	0.780	2927	3.14 × 10^−7^	420	125.6

**Table 2:** Comparison of different electrodes.

Electrode Type	Contact Impedance	Adhesion	Reusability
Rubber dry electrode [30]	50 kΩ·cm^2^	EEG hat or bandage needed	Yes
Conductive fabric electrode [26]	200 kΩ·cm^2^	Bndage needed	Yes
Gel electrode [31]	20 kΩ·cm^2^	Etra tape needed	No
Temporary tattoo electrode [32]	17 × 10^3^ kΩ·cm^2^	Yes	No
Bacterial cellulose electrode	19 kΩ·cm^2^	Yes	No

## Data Availability

Data sharing not applicable.
